# Comparison of clinical outcomes for different morphological scores of D5 and D6 blastocysts in the frozen-thawed cycle

**DOI:** 10.1186/s12884-023-05415-w

**Published:** 2023-02-06

**Authors:** Yaping Jiang, Rui Jiang, Hui He, Xinling Ren, Qiong Yu, Lei Jin

**Affiliations:** 1 Department of Reproductive Medicine Center, Tongji Hospital, Tongji Medical College, Huazhong University of Science and Technology, Wuhan, Hubei PR China; 2 Laboratory of Clinical Immunology, Wuhan No. 1 Hospital, Tongji Medical College, Huazhong University of Science and Technology, Wuhan, Hubei PR China

**Keywords:** Embryo selection, Embryo development speed, Embryo morphology score, Frozen embryo transfer, Pregnancy outcomes

## Abstract

**Background:**

Both embryo development speed and embryo morphology score played a significant role in frozen-thawed embryo transfer cycle (FET) outcomes. Most of the literature indicates that D5 embryos performed better than D6 embryos, although a few also indicate that there is no difference in clinical outcomes between D5 and D6 embryos. Clinically, D5 embryos are preferred for equal morphological scores. But how to choose embryos when the morphological score of D6 embryos is better than D5?

**Methods:**

A retrospective study including 8199 frozen-thawed embryo transfers (FETs) was conducted to analyze patients who underwent IVF-FET from January 2018 to December 2020. Patients were divided into 8 groups according to the rate of embryonic development and morphological scores to compare pregnancy outcomes. We further compared clinical pregnancy outcomes and neonatal outcomes between BC embryos on day 5 (D5) and BA/BB embryos on day 6 (D6).

**Results:**

Our study found no difference in clinical pregnancy rate (CPR) and live birth rate (LBR) between AA/AB blastocysts in D5 or D6 frozen blastocysts. However, for BA/BB/BC blastocysts, embryonic pregnancy outcome was significantly better in D5 than in D6. In our further analysis and comparison of BC embryos in D5 and BA/BB embryos in D6, we found no difference in clinical pregnancy outcomes and neonatal outcomes, but D6 BA/BB embryos had a higher rate of miscarriage. After adjusting for confounding factors, none of the indicators differed between groups.

**Conclusion:**

Our study provides suggestions for embryo selection: AA/AB embryos are preferred, regardless of the embryo development day, and the second choice is BA or BB embryos on D5. BA/BB embryos in D6 had a higher miscarriage rate than BC embryos in D5 but were not statistically significant after adjusting for confounding factors.

**Supplementary Information:**

The online version contains supplementary material available at 10.1186/s12884-023-05415-w.

## Introduction

The blastocyst embryo transfer is becoming increasingly popular because it improves the chances of pregnancy and shortens the time to pregnancy through better embryo selection [1–3]. Transferring blastocysts has been shown to increase pregnancy rates and live birth rates compared to cleavage-stage embryos [4]. In addition, this strategy encourages singleton pregnancies, thereby reducing the rate of multiple pregnancies and the complications and costs associated with them [5, 6].

Post-fertilization embryos usually form blastocysts by D5 in culture, but slower embryos can form blastocysts by D6 or later. Many studies have attempted to observe if there are differences in ART outcomes for blastocysts developing on D5 and D6, but with conflicting results. In fresh cycles, there is already evidence of superior clinical results with D5 transfers compared with D6 transfers [7]. Barrenetxea et al. demonstrated that there is a higher pregnancy rate with D5 embryo transfers after fertilization when compared with D6 blastocyst transfers in fresh cycles [8].

With the widespread use of vitrification protocols, many studies have shown that in FET cycles, D5 blastocysts performed better than D6 blastocysts [9–14]. Specifically, Bourdon et al. performed a meta-analysis of FET and found that D5 embryo transfers had a higher clinical pregnancy rate (CPR) and live birth rate (LBR) than D6 embryo transfers [15].

However, several publications show no difference in pregnancy rates between D5 and D6. In a study by Shapiro et al., there was no significant difference between blastocysts cryopreserved on D5 or D6 in FET cycles [16]. In a meta-analysis published by Sunkara et al., similar results were obtained for blastocysts frozen on D5 and D6 transferred from embryos at the same developmental stage [17]. An article indicates that clinical and pregnancy rates for cryopreserved blastocysts are comparable after vitrification on D5 and D6 [18].

Besides the embryo developmental stage, the embryo morphology score is also important to the success of IVF treatment. Traditionally, standard morphological assessments have been used to predict the implantation potential of embryos [19]. Better morphological scores were associated with higher rates of euploidy and therefore higher implantation rate (IR) [20]. There is evidence that morphological grading allows the selection of blastocysts with high implantation potential even in euploid embryos [21]. Whether transferred fresh or vitrified-warmed, a high-quality blastocyst has been proven to increase the success rate [22–24].

Embryo selection is one of the key factors in increasing pregnancy chances and shortening pregnancy time. For embryologists, choosing embryos with the best implantation potential is a constant challenge. Although research has been conducted for decades, the most important methods for selecting embryos for transfer and cryopreservation are based on their developmental speed and morphological scores [25, 26]. Therefore, we designed this retrospective study to compare the pregnancy outcomes of FET cycles between D5 and D6 at different grades of embryo quality. Additionally, we further compared the ART outcomes of FET cycles between BC embryos in D5 and BA/BB embryos in D6. We expect that our results will provide further guidance in selecting the best blastocysts and help embryologists to implement better embryo transfer strategies.

## Materials and methods

### Study design and patient selection

We conducted a retrospective analysis of freeze-thaw cycles from January 2018 to December 2020 at Tongji Hospital, Tongji Medical College, Huazhong University of Science and Technology. Women who underwent FETs with a single blastocyst transfer were included in the study. Couples who underwent pre-implantation genetic test (PGT), in vitro maturation (IVM) or egg thawing were excluded. Couples undergoing repeated cryopreservation were also excluded due to data from our center suggesting that embryos in the repeated cryopreservation group had significantly lower IR, CPR, and LBR [27]. 

### Human blastocyst culture

IVF or ICSI was performed depending on previous fertilization history and sperm parameters. The presence of two nuclei (2PN) is the marker for fertilization, which was examined 16 h after insemination/injection. The zygotes were cultured in G1-plus medium (Vitrolife, Sweden) to the cleavage stage until D3. Usually, one or two D3 embryos are selected for transfer or frozen on day 3 of fertilization (6 cells and above, fragmentation less than or equal to 20). The remaining embryos are transferred to G2-plus medium (Vitrolife, Sweden) culture and continue to be cultured until D5 or D6. Before freezing on day 5 or 6, blastocysts were graded according to the Gardner grading system [19]. Firstly, blastocysts are classified into 6 stages according to their degree of expansion. Then, stage 3 to stage 6 blastocysts were further evaluated for the quality of their inner cell mass (ICM) and trophoblast ectoderm (TE). We performed freezing when the Gardner score is greater than or equal to 3 BC. Our center has 10 experienced embryologists involved in blastocyst quality assessment. And, we conduct embryo scoring criteria quality control meetings every 3 months to reduce subjective differences in individual evaluation of embryos.

### Vitrification and warming procedure

The vitrification process was performed using a commercial kit (Kitazato Company, Japan). The blastocyst was drilled with laser irradiation at the junction of trophoblast cells far from the inner cell mass before freezing, and the cavity was allowed to shrink before freezing. The shrunken blastocyst was transferred to equilibration solution for 5–8 min. Then, the embryos were exposed to the vitrification solution for 45–60 s. Embryos were loaded onto the Cryotop (Kitazato, Japan) with a very small volume of vitrification solution, after which they were immediately immersed in liquid nitrogen.

A Vitrification warming kit (Kitazato, Japan) was preheated at room temperature before warming. The embryos were immediately placed in pre-equilibrated thawing solution (TS) for 1 min and dilution solution (DS) for 3 min according to the instructions of the warming kit, then washed twice for 5 min each in Washing Solution 1 (WS1) and Washing Solution 2 (WS2). They were transferred to G2 blastocyst culture medium (Vitrolife, Gothenburg, Sweden) and incubated with 5% CO2 at 37 °C for further transfer.

### Outcome measures

HCG serum pregnancy tests were performed 12 to 14 days following blastocyst transfer, and clinical pregnancy was defined as the detection of a gestational sac with fetal heart pulsations on ultrasound scanning 4 to 5 weeks following blastocyst transfer. Live birth rate was defined as the delivery of a viable infant after 24 weeks of gestation. Miscarriage was defined as the spontaneous loss of pregnancy before 20 weeks of gestation. Ectopic pregnancies were defined as clinical pregnancies occurring outside the uterine cavity. The neonates-related characteristics comprised cesarean section, gender, gestational age, and birth weight. Preterm delivery is defined as a gestational age of fewer than 37 weeks. High birth weight, low birth weight, and very low birth weight were defined as birthweight ≥4000 g, < 2500 g, and < 1500 g, respectively.

### Statistical analysis

The data were analyzed using version SPSS26.0 (IBM, USA). Continuous data are given as means and standard deviations, while categorical variables are expressed as percentages. The t-test was used to evaluate differences between groups for continuous data, and Pearson’s chi-square test was used for categorical variables. A *P*-value of less than 0.05 was considered statistically significant in two-tailed hypothesis tests.

## Results

**Fig. 1:**
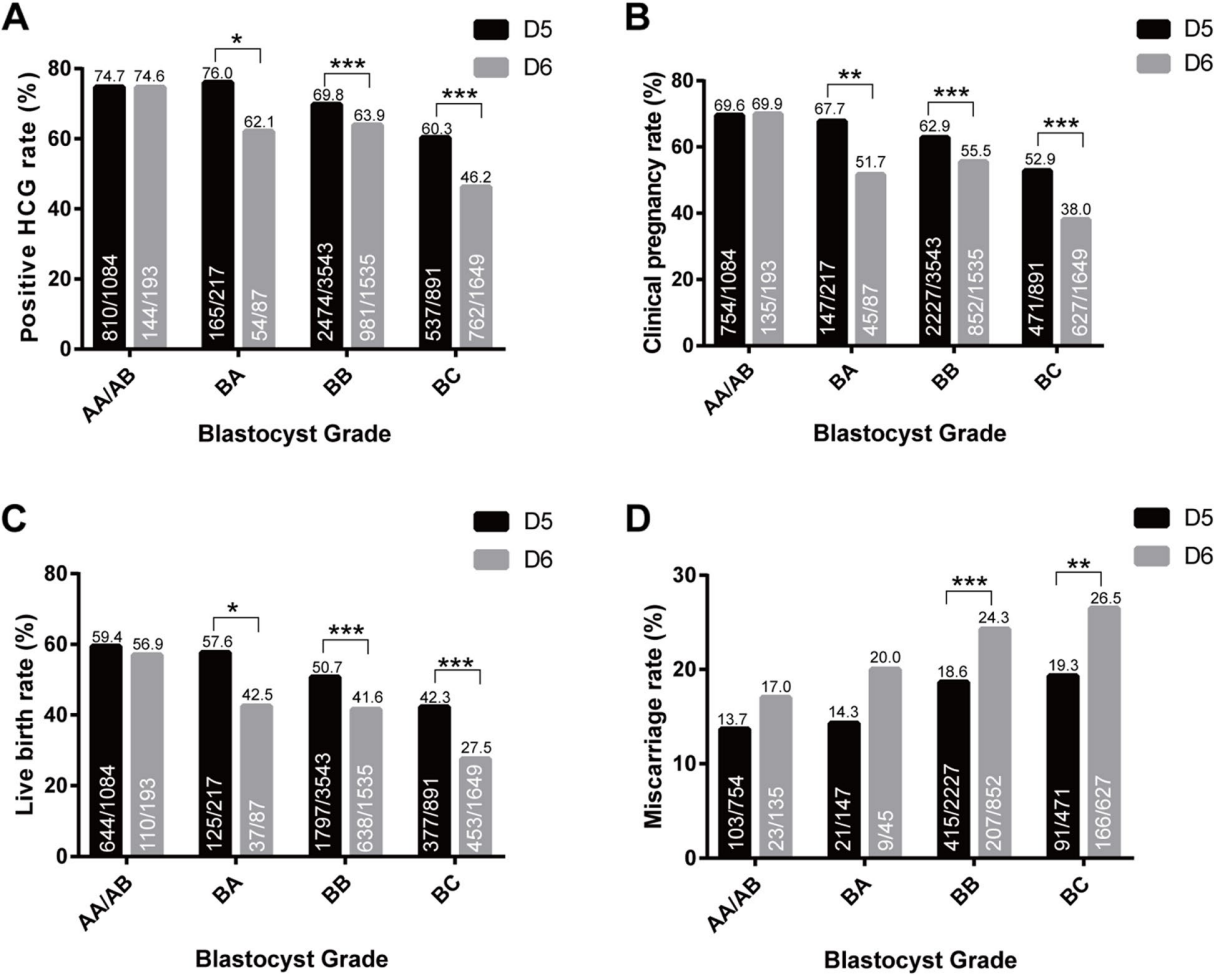
Pregnancy outcomes in D5 and D6 between different groups of blastocysts

Figure 1 compares pregnancy outcomes in D5 and D6 between different groups of blastocysts. The characteristics of patients are presented in Supplementary Tables 1 and 2. For the AA/AB group, the positive HCG rate and CPR were not significantly different between D5 and D6 (74.7 vs. 74.6%, 69.6 vs. 69.9%). The D5 group had a higher LHR than D6 (59.4 vs. 56.9%), and the D5 group had a lower miscarriage rate (13.7 vs. 17.0%), but the difference was not statistically significant. For the BA group, the positive HCG rate (76.0 vs. 62.1%), CPR (67.7 vs. 51.7%), and LHR (57.6 vs. 42.5%) were significantly higher in D5 than in D6 (*P* < 0.05). The miscarriage rate was lower in D5 than in D6 (14.3 vs. 20.0%), but the difference was not statistically significant. For the BB group, the positive HCG rate (69.8 vs. 63.9%), CPR (62.9 vs. 55.5%), and LHR (50.7 vs. 41.6%) were higher for D5 than for D6, and the miscarriage rate was lower than for D6 (18.6 vs. 24.3%), all with statistically significant differences (*P* < 0.05). Similarly, for the BC group, the positive HCG rate (60.3 vs. 46.2%), CPR rate (52.9 vs. 38.0%), and LHR rate (42.3 vs. 27.5%) were higher in D5 than in D6, and the miscarriage rate (19.3 vs. 26.5%) was lower than in D6, all with statistically significant differences (*P* < 0.05).

This indicates that there was no difference in pregnancy outcome between D5 and D6 in high quality blastocysts (AA/AB), while embryos from D5 were significantly better than those from D6 in both good quality blastocysts (BB/BA) and poor quality blastocysts (BC). The results did not change after correcting for confounding factors, including age, BMI, endometrial thickness, number of oocytes retrieved, infertility type, duration of infertility, fertilization method, endometrial preparation program, and embryo development stages (Table 1). However, in the FET cycle, when the morphological score of D6 embryos is better than that of D5, we need to decide whether to thaw D5 embryos of poor quality or D6 embryos of good quality. To assist in making this decision, we then went on to analyze D5 BC and D6 BA/BB embryos.

**Table 1:** Results of logistic regression analysis of pregnancy outcomes

	D5 AA/AB (*n* = 1084)	D6 AA/AB (*n* = 193)	Unadjusted OR (95% CI)	Adjusted OR (95% CI)
Positive HCG rate	74.72 (810/1084)	74.61 (144/193)	1.007 (0.708,1.432)	0.897 (0.600,1.340)
Clinical pregnancy rate	69.56 (754/1084)	69.95 (135/193)	0.982 (0.703,1.371)	0.863 (0.593,1.256)
Miscarriage rate	13.66 (103/754)	17.04 (23/135)	0.778 (0.481，1.259)	0.776 (0.455,1.321)
Live birth rate	59.41 (644/1084)	56.99 (110/193)	1.103 (0.810,1.504)	0.982 (0.696,1.386)
	D5 BA (*n* = 217)	D6 BA (*n* = 87)	Unadjusted OR (95% CI)	Adjusted OR (95% CI)
Positive HCG rate	76.04 (165/217)	62.07 (54/87)	2.136 (1.257,3.628)	2.302 (1.262,4.202)
Clinical pregnancy rate	67.74 (147/217)	51.72 (45/87)	1.960 (1.180,3.257)	2.145 (1.205,3.815)
Miscarriage rate	14.29 (21/147)	20.00 (9/45)	0.929 (0.407,2.116)	0.928 (0.365,2.360)
Live birth rate	57.60 (125/217)	42.53 (37/87)	1.925 (1.162,3.188)	2.105 (1.182,3.748)
	D5 BB (*n* = 3543)	D6 BB (*n* = 1535)	Unadjusted OR (95% CI)	Adjusted OR (95% CI)
Positive HCG rate	69.83 (2474/3543)	63.91 (981/1535)	1.303 (1.148,1.479)	1.421 (1.228,1.645)
Clinical pregnancy rate	62.86 (2227/3543)	55.50 (852/1535)	1.354 (1.198,1.529)	1.440 (1.251,1.656)
Miscarriage rate	18.63 (415/2227)	24.30 (207/852)	0.716 (0.592,0.865)	0.769 (0.619,0.954)
Live birth rate	50.72 (1797/3543)	41.56 (638/1535)	1.439 (1.274,1.624)	1.462 (1.273,1.681)
	D5 BC (*n* = 891)	D6 BC (*n* = 1649)	Unadjusted OR (95% CI)	Adjusted OR (95% CI)
Positive HCG rate	60.27 (537/891)	46.21 (762/1649)	1.747 (1.479,2.064)	1.909 (1.592,2.289)
Clinical pregnancy rate	52.86 (471/891)	38.02 (627/1649)	1.809 (1.532,2.136)	1.984 (1.655,2.379)
Miscarriage rate	19.32 (91/471)	26.48 (166/627)	0.679 (0.507,0.909)	0.673 (0.490,0.924)
Live birth rate	42.31(377/891)	27.47 (453/1649)	1.907 (1.604,2.266)	2.066 (1.710,2.498)

As shown in Table 2, the baseline characteristics of the study population are listed. The maternal age, body mass index (BMI), the thickness of the endometrium, FSH, and number of oocytes retrieved was comparable between the two groups (D5 BC and D6 BA/BB; *p* > 0.05). Additionally, there was no difference between the groups in the proportion of infertility types, durations of infertility, and endometrial preparation programs (*p* > 0.05). There was a significant difference in the fertilization method and embryo development stages (*p* < 0.001), grade 4 blastocysts are more likely to be found in the D6 BA/BB group.

**Table 2:** Baseline clinical characteristics of the study population

	D5 BC	D6 BA/BB	*P* value
Number of FET cycle	*n* = 891	*n* = 1622	
Maternal age (years), mean ± SD	31.97 ± 4.58	31.69 ± 4.30	0.125
Maternal BMI, mean ± SD	21.49 ± 3.15	22.32 ± 3.09	0.434
Endometrial thickness (mm), mean ± SD	9.35 ± 1.52	9.30 ± 1.47	0.434
FSH	7.43 ± 2.35	7.43 ± 2.13	0.977
Number of oocytes retrieved, mean ± SD	11.45 ± 6.90	11.84 ± 7.76	0.218
Type of infertility, n (%)			0.143
Primary infertility	63.64 (567/891)	60.67 (984/1622)	
Second infertility	36.36 (324/891)	39.33 (638/1622)	
Duration of infertility (years), mean ± SD	3.54 ± 2.50	3.48 ± 2.36	0.819
Fertilization method, n (%)			<0.001
IVF	61.28 (546/891)	54.25 (880/1622)	
ICSI	33.67 (300/891)	37.18 (603/1622)	
IVF + ICSI	5.05 (45/891)	8.57 (139/1622)	
Endometrial preparation program, n (%)			0.849
Artificial cycle	92.70 (826/891)	92.91 (1507/1622)	
Natural cycle	7.30 (65/891)	7.09 (115/1622)	
Embryo developmental stages			<0.001
3	37.71 (336/891)	8.82 (143/1622)	
4	61.62 (549/891)	77.87 (1263/1622)	
5	0.34 (3/891)	6.23 (101/1622)	
6	0.34 (3/891)	7.09 (115/1622)	

Table 3 presents the pregnancy outcomes of the study population. There was no significant difference in the positive HCG rate, clinical pregnancy rate, ectopic pregnancy rate, and live birth rate between the two groups, but the miscarriage rate ([OR] 1.309, 95% confidence interval [CI] 1.048–1.636) in the D6 BA/BB group was higher. After adjusting for possible confounding factors, the miscarriage rate was not statistically significant ([OR] 1.233, 95% confidence interval [CI] 0.958–1.561).

**Table 3:** Results of logistic regression analysis of pregnancy outcomes

	D5 BC (*n* = 891)	D6 BA/BB (*n* = 1622)	Unadjusted OR (95% CI)	Adjusted OR (95% CI)
Positive HCG rate	60.27 (537/891)	63.81 (1035/1622)	1.156 (0.977,1.368)	1.037 (0.860,1.251)
Clinical pregnancy rate	52.86 (471/891)	55.30 (897/1622)	1.103 (0.936,1.300)	0.984 (0.820,1.182)
Miscarriage rate	19.32 (91/471)	24.08 (216/897)	1.309 (1.048,1.636)	1.233 (0.958,1.561)
Ectopic pregnancy rate	0.63 (3/471)	0.67 (6/897)	1.099 (0.274,4.405)	0.484 (0.109,2.145)
Live birth rate	42.31 (377/891)	41.62 (675/1622)	0.969 (0.821,1.144)	0.898 (0.747,1.081)

The neonatal outcomes of all singletons born after FET were compared between the two groups (Table 4). There was no significant difference in gestational age and birth weight between the two groups. There were no differences in the outcomes of preterm births, low birth weights, very low birth weights, and high birth weights between the groups. Table 5 displays the result from a multivariable logistic regression analysis of the relationship between the two groups. After multivariable linear regression was conducted and other potential confounders were adjusted, there were no significant differences between the groups in neonatal outcomes (Table 5). According to the multivariable linear regression, the gestational age and birth weight did not differ significantly between the two groups (Table 6).

**Table 4:** Neonatal outcomes of singleton live births in frozen embryo transfer

	D5 BC (*n* = 373)	D6 BA/BB (*n* = 667)	*P* value
Gestational age (wk), mean ± SD	38.44 ± 1.80	38.58 ± 2.17	0.247
Premature (< 37 weeks), n (%)	10.46 (39/373)	8.55 (57/667)	0.307
Birth weight (g), mean ± SD	3319.96 ± 525.838	3319.96 ± 525.838	0.141
Low birth weight (< 2500 g), n (%)	4.56(17/373)	4.05 (27/667)	0.695
Very low birth weight (< 1500 g), n (%)	0.80(3/373)	0.45 (3/667)	0.469
High birth weight (> 4000 g), n (%)	7.77(29/373)	7.65 (51/667)	0.940

**Table 5:** Results of logistic regression analysis of neonatal outcomes in singletons

Variable	D5 BC vs. D6 BA/BB	
	Unadjusted OR (95% CI)	Adjusted OR (95% CI)
Premature (< 37 weeks), n (%)	0.800 (0.521,1.228)	0.732 (0.454,1.182)
Low birth weight (< 2500 g), n (%)	0.883 (0.475,1.643)	0.907 (0.459,1.794)
Very low birth weight (< 1500 g), n (%)	0.557 (0.112,2.775)	0.261 (0.040,1.711)
High birth weight (> 4000 g), n (%)	0.982 (0.611,1.578)	1.014 (0.602,1.710)

**Table 6:** Multiplicative regression results for gestation age and birth weight in singletons

Variable	D5 BC vs. D6 BA/BB		
	β	Standard error	*P* value
Gestational age	−0.148	0.131	0.257
Birth weight	−48.335	32.439	0.137

## Discussion

We found there was no difference in pregnancy outcome between D5 and D6 in high quality blastocysts (AA/AB), but for good quality blastocysts (BA/BB) and poor quality blastocysts (BC), the pregnancy outcome was significantly higher for blastocysts at D5 than at D6. However, how to select the optimal blastocyst for thawing during the FET cycle when the morphological score of D6 blastocysts is good quality (BA/BB), and the score of D5 blastocysts is poor quality (BC)? To answer this question, we further analyzed and comparison of D5 BC and D6 BA/BB embryos, we found that D6 BA/BB had a higher pregnancy rate than D5 BC, but D6 BA/BB also had a higher miscarriage rate than D5 BC, so there was no difference in the live birth outcome. Although confounding factors were adjusted for, none of the indicators differed between groups.

Researchers have attempted to investigate whether D5 or D6 blastocysts would produce different ART outcomes. With the increasing use of vitrification, more studies have shown that D5 embryos have better ART outcomes than D6 embryos during FET. Our findings are consistent with this conclusion, D5 embryos have significantly higher CPR and LBR than D6 embryos. Nevertheless, our study found no difference between D5 and D6 in CPR or LBR when the embryo score was AA/AB. This conclusion is supported by several studies. A study by Yang et al. showed that high quality D6 blastocysts had similar pregnancy outcomes to high quality D5 blastocysts in freeze-thaw cycles [11]. According to Shen et al., the embryo development day (D5 or D6) did not affect LBR for AA/AB/BA blastocysts, but embryos on D5 had significantly higher LBR than embryos on D6 for BB/BC/CB blastocysts [28].

There is also some literature showing that D5 blastocysts have better clinical outcomes than D6 blastocysts in FET cycles, regardless of blastocyst score. As in Haas et al., good-quality blastocysts on D5 and D6 are compared [10]. Based on 791 frozen embryo transfers, the researchers concluded that the CPR was significantly lower for embryos frozen on D6 than for those frozen on D5. Another study by Ferreux et al. showed that LBR with FET was significantly higher in D5 than in D6 embryos, regardless of embryo quality [12]. But in these researches, an embryo of good quality is defined as an embryo≥3BB, which includes AA, AB, BA, and BB. It is noteworthy that Irani et al. reported more favorable clinical outcomes after D5 FET than D6 FET in similarly graded frozen euploid blastocysts [29]. The results of our study revealed a significantly higher CPR and LBR than D6 for BA/BB/BC grade blastocysts, which was consistent with many previous studies [10, 12, 13, 15, 29].

It has been demonstrated that D5 transfers are more clinically effective than D6 transfers in fresh cycles [7, 8, 30]. These observations may be explained by delayed embryo growth and a displaced window of implantation. FET cycles allow for a better understanding of the direct impact of embryo developmental rate on embryo implantation potential, as FET can control endometrial factors. By analyzing fresh and FET cycles, Shapiro et al. found that blastocysts implanted at D5 had higher CPR than those implanted at D6 in the fresh cycle, but produced similar results in the FET cycle [16]. Thus, the authors conclude that D5 and D6 embryos exhibit similar developmental potential and that the differences in pregnancy outcomes observed in fresh cycles may be due to asynchrony between endometrium. In frozen blastocyst transfer, there should be no difference in pregnancy rates between D5 and D6 if only the endometrium is asynchronous. The fact that this did not happen indirectly implies that there are other reasons for the higher implantation potential of D5 blastocysts.

One possible explanation for the higher pregnancy rate of blastocysts on D5 compared to D6 stems from the higher aneuploidy rate among D6 embryos. There is some evidence that the differences in the outcome of blastocysts on D5 and D6 may be explained by data on PGS and blastocyst formation days (D5 and D6) in FET cycles. The D6 embryos with delayed development are more likely to be aneuploid [31, 32]. Minasi et al. found shorter blastocyst formation time and higher quality and amplification grade of TE and ICM in euploid embryos [20]. Nevertheless, Iran et al. [29] compared D5 and D6 euploid blastocysts and found that D5 embryos had a superior implantation potential. In addition, the authors speculate that other epigenetic or metabolic differences may be contributing to the different implantation potential between D5 and D6 embryos. Although euploid embryos have higher implantation potential, 30–40% of euploid embryos fail to produce live births [29]. In these cases, implantation failure can be caused by pathologies of the endometrium, embryo endometrial dyssynchrony, thrombophilia, hydrosalpinx, or the presence of an immunological problem [33]. In addition, a variety of molecules involved in the regulation of implantation, zona pellucida, oocyte quality, sperm quality, granulosa cells, age, and other factors can also affect the success of implantation [34–36].

Additionally, our results show that in BB and BC groups, although the morphological scores of embryos are the same, the miscarriage rate of D6 is higher than that of D5. After correcting confounding factors, the miscarriage rate is still statistically different. Multivariate logistic regression analysis suggests that embryo development days, age, BMI, and endometrial thickness are related to the miscarriage rate. According to a meta-analysis, there was no significant difference in miscarriage rates between blastocyst transfers from D5 and D6 blastocysts in FET cycles [17]. Furthermore, Ferreux et al. found similar miscarriage rates in D5 and D6 FET cycles [12]. In contrast, in the study by Wang et al., the miscarriage rate of D6 embryos was higher than that of D5 embryos, although the difference was not statistically significant [37]. According to Yang et al., miscarriage rates were higher in D6 blastocysts than in D5 blastocysts [11]. Moreover, the risk of miscarriage was associated with the day of blastocyst expansion, and the risk of miscarriage was higher in D6 ET than in D5 ET [15].

This study has some limitations. First, AC, CA, and CB embryos were not included in this study due to the small sample size. Second, the findings are likely to be biased because the sample size of each group is heterogeneous. Further studies are needed to further confirm the findings of this study. Additionally, the embryo morphology scoring method adopted in this study is subjective to some extent. Morphological scoring is currently used for screening embryos, and the commonly used scoring scheme for blastocysts is that devised by Gardner et al. in 2000 [19]. Considering the inherent subjectivity of morphological assessment, it would be useful to assess observer variability [38]. In addition to routine morphological assessment, there are several invasive or non-invasive embryo selection methods, such as preimplantation genetic testing, morphological kinetics, proteomics, metabonomics, oxygen consumption, and measurement of oxidative stress in culture medium [39]. Morphological dynamics is a method based on timelapse technology and continuous monitoring of embryos [40]. The models for artificial intelligence to select optimal embryos are currently being optimized and refined [41, 42].

Our study provides suggestions for embryo selection: AA/AB embryos are preferred, regardless of the embryo development speed, and the second choice is BA or BB embryos on D5. BA/BB embryos in D6 had a higher miscarriage rate than BC embryos in D5 but were not statistically significant after adjusting for confounding factors.

## Supplementary Information

**Additional file 1: Supplementary Table 1.** Baseline Clinical characteristics of the study population. **Supplementary Table 2.** Baseline Clinical characteristics of the study population.

## Data Availability

The datasets used and/or analyzed during the current study are available from the corresponding author on reasonable request.
