# Supplementary material for: Comparison of clinical outcomes for different morphological scores of D5 and D6 blastocysts in the frozen-thawed cycle
Source: BMC Pregnancy Childbirth. 2023 Feb 6;23:97. doi: 10.1186/s12884-023-05415-w (PMC9900991; doi:10.1186/s12884-023-05415-w)
Supplement: Supplementary file 1 — Additional file 1: Supplementary Table 1. Baseline Clinical characteristics of the study population. Supplementary Table 2. Baseline Clinical characteristics of the study population. [file 12884_2023_5415_MOESM1_ESM.doc]

|  | D5 AA/AB | D6 AA/AB | P value | D5 BA | D6 BA | P value |
| --- | --- | --- | --- | --- | --- | --- |
| Number of FET cycle | n=1084 | n=193 |  | n=217 | n=87 |  |
| Maternal age (years), mean±SD | 31.70±3.79 | 31.76±4.36 | 0.835 | 30.91±3.95 | 31.17±3.67 | 0.597 |
| Maternal BMI,mean±SD | 21.49±2.96 | 21.49±2.70 | 0.987 | 21.43±2.67 | 21.05±2.19 | 0.236 |
| Endometrial thickness (mm),mean±SD | 9.41±1.51 | 9.36±1.50 | 0.695 | 9.38±1.64 | 9.31±1.52 | 0.713 |
| FSH | 7.15±1.84 | 7.30±1.92 | 0.303 | 7.53±1.78 | 7.24±1.87 | 0.646 |
| Number of oocytes retrieved, mean ±SD | 17.78±7.96 | 15.85±7.06 | 0.002 | 17.36±8.16 | 16.17±7.57 | 0.243 |
| Type of infertility, n (%) |  |  | 0.806 |  |  | 0.251 |
| Primary infertility | 68.54  (743/1084) | 69.43  (134/193) |  | 64.52  (140/217) | 57.47  (50/87) |  |
| Second infertility | 31.46  (341/1084) | 30.57  (59/193) |  | 35.48  (77/217) | 42.53  (37/87) |  |
| Duration of infertility (years),mean±SD | 3.10±2.17 | 3.46±2.40 | 0.044 | 2.98±2.27 | 3.31±2.37 | 0.270 |
| Fertilization method, n (%) |  |  | 0.001 |  |  | 0.357 |
| IVF | 71.78  (778/1084) | 62.18  (120/193) |  | 63.13  (137/217) | 63.22  (55/87) |  |
| ICSI | 25.18  (273/1084) | 30.05  (58/193) |  | 29.95  (65/217) | 25.29  (22/87) |  |
| IVF+ICSI | 3.04  (33/1084) | 7.77  (15/193) |  | 6.91  (15/217) | 11.49  (10/87) |  |
| Endometrial preparation program, n (%) |  |  | 0.775 |  |  | 0.379 |
| artificial cycle | 95.30  (1033/1084) | 94.82  (183/193) |  | 91.24  (198/217) | 94.25  (82/87) |  |
| natural cycle | 4.70  (51/1084) | 5.18  (10/193) |  | 8.76  (19/217) | 5.75  (5/87) |  |
| Embryo developmental stages |  |  | ＜0.001 |  |  | 0.026 |
| 3 | 7.84  (85/1084) | 1.55  (3/193) |  | 1.84  (4/217) | 2.30  (2/87) |  |
| 4 | 91.14  (988/1084) | 89.64  (173/193) |  | 96.77  (210/217) | 89.66  (78/87) |  |
| 5 | 0.83  (9/1084) | 5.18  (10/193) |  | 0.46  (1/217) | 4.60  (4/87) |  |
| 6 | 0.18  (2/1084) | 3.63  (7/193) |  | 0.92  (2/217) | 3.45  (3/87) |  |

**Supplementary Table 1. Baseline Clinical characteristics of the study population**

**Supplementary Table 2**. Baseline Clinical characteristics of the study population

|  | D5 BB | D6 BB | P value | D5 BC | D6 BC | P value |
| --- | --- | --- | --- | --- | --- | --- |
| Number of FET cycle | n=3543 | n=1535 |  | n=891 | n=1649 |  |
| Maternal age (years), mean±SD | 31.32±4.24 | 31.72±4.33 | 0.002 | 31.97±4.58 | 32.47±4.80 | 0.012 |
| Maternal BMI,mean±SD | 21.81±3.09 | 22.28±3.12 | 0.002 | 22.49±3.15 | 21.75±2.96 | ＜0.001 |
| Endometrial thickness (mm),mean±SD | 9.33±1.46 | 9.30±1.46 | 0.546 | 9.35±1.52 | 9.32±1.48 | 0.593 |
| FSH | 7.32±2.40 | 7.44±2.13 | 0.091 | 7.43±2.35 | 7.65±2.82 | 0.050 |
| Number of oocytes retrieved, mean ±SD | 16.12±7.65 | 11.59±7.71 | ＜0.001 | 11.45±6.90 | 12.29±6.38 | 0.009 |
| Type of infertility, n (%) |  |  | 0.079 |  |  | 0.261 |
| Primary infertility | 63.70  (2257/3543) | 61.11 (938/1535) |  | 63.64  (567/891) | 61.37  (1012/1649) |  |
| Second infertility | 36.30  (1286/3543) | 38.89 (597/1535) |  | 36.36  (324/891) | 38.63  (637/1649) |  |
| Duration of infertility (years),mean±SD | 3.26±2.32 | 3.49±2.36 | 0.001 | 3.54±2.50 | 3.23±2.43 | 0.009 |
| Fertilization method, n (%) |  |  | ＜0.001 |  |  | 0.001 |
| IVF | 66.92  (2371/3543) | 53.68 (824/1535) |  | 61.28  (546/891) | 60.52 (998/1649) |  |
| ICSI | 27.24  (965/3543) | 37.92 (582/1535) |  | 33.67  (300/891) | 30.44 (502/1649) |  |
| IVF+ICSI | 5.84  (207/3543) | 8.40 (129/1535) |  | 5.05  (45/891) | 9.04  (149/1649) |  |
| Endometrial preparation program, n (%) |  |  | 0.218 |  |  | 0.852 |
| artificial cycle | 93.76  (3322/3543) | 92.83 (1425/1535) |  | 92.70  (826/891) | 92.90 (1532/1649) |  |
| natural cycle | 6.24  (221/3543) | 7.17 (110/1535) |  | 7.30  (65/891) | 7.10  (117/1649) |  |
| Embryo developmental stages |  |  | ＜0.001 |  |  | ＜0.001 |
| 3 | 26.87  (952/3543) | 9.19 (141/1535) |  | 37.71  (336/891) | 16.68  (275/1649) |  |
| 4 | 71.69  (2540/3543) | 77.20 (1185/1535) |  | 61.62  (549/891) | 75.02 (1237/1649) |  |
| 5 | 0.82  (29/3543) | 6.32 (97/1535) |  | 0.34  (3/891) | 5.22  (86/1649) |  |
| 6 | 0.62  (22/3543) | 7.30 (112/1535) |  | 0.34  (3/891) | 3.09  (51/1649) |  |
